# Editorial: Health Implications of Dietary Fibers and Bioactive Carbohydrates From Natural Resources

**DOI:** 10.1002/fsn3.70208

**Published:** 2025-05-06

**Authors:** Bin Du, Baojun Xu

**Affiliations:** ^1^ Hebei Key Laboratory of Natural Products Activity Components and Function Hebei Normal University of Science and Technology Qinhuangdao Hebei People's Republic of China; ^2^ Food Science and Technology Program, Department of Life Sciences Beijing Normal‐Hong Kong Baptist University Zhuhai People's Republic of China

This Research Topic collected the latest findings on the biological activity and potential applications of dietary fiber DF and polysaccharide. In this special e‐collection, there are 14 papers covering the above‐mentioned aspects, including two reviews, two systematic reviews and meta‐analyses, eight research papers, and two clinical trials. The results of the above‐mentioned studies will fill the gap between the knowledge of significant health implications of DFs and bioactive carbohydrates and their underlying cellular signaling and molecular mechanisms.

Natural resources are abundant in dietary fiber and bioactive carbohydrates, including bioactive polysaccharides and oligosaccharides (Stephen et al. 2017). Extensive investigations have focused on the gut microbiota modulation effects of various dietary fibers, such as cellulose, oligosaccharide, pectin, lignin, type‐II and type‐IV resistant starches, colloid, and complex arabinoxylan (Deehan et al. 2022). As a result of the increased attention to the sustainability of natural resources, efforts have been made to use fibers in different bio‐industries. There are some preliminary studies on the correlation between dietary fiber and biological activities. However, the underlying mechanism beyond them is not clear.

This Research Topic collected the latest findings on therapeutic potentials and industrial uses of dietary fiber and polysaccharide from natural resources, such as mangiferin, inulin‐type fructans, silymarin, Chinese yam bulbils polysaccharides, *Dendrobium officinale* leaf polysaccharides, hawthorn pectin, *Polygonatum odoratum* polysaccharide, fiber, guar gum, chitosan, protein isolates–konjac glucomannan compound, purslane, pumpkin, and quinoa.

In this special issue, there are 14 manuscripts, including the above‐mentioned contents, including two reviews, two systematic reviews and meta‐analysis, eight research papers, and two clinical trials. Plant‐derived polysaccharides, in particular, have garnered attention in recent years. There are four papers covering the plants polysaccharide research. Zhou et al. investigated that polysaccharides from Chinese yam bulbils exhibit anti‐fatigue effects in mice, correlating with enhanced hepatic glycogen storage and antioxidant enzyme vitality. Molecular weight variations were proposed as a key determinant of their efficacy (Zhou et al. 2023).

Similarly, Xiong et al. (2023) characterized a low‐molecular‐weight polysaccharide (LDOP‐A) from Dendrobium officinale leaves, revealing its partial digestibility and short‐chain fatty acid production during colonic fermentation.

Liu et al. (2023) further highlighted the anti‐inflammatory role of *Polygonatum odoratum* polysaccharide in mitigating lung injury via gut microbiota modulation and macrophage regulation.

Pectin has been widely used as gelling agents, emulsifiers, glazing agents, stabilizers, and thickeners in food industries. In one study, pectin research revealed that ethanol fractionation alters physicochemical properties, with CAHP60 displaying superior thermal stability and emulsifying capacity (Wang et al. 2023).

Meanwhile, Iqbal et al. (2023) synthesized evidence on mangiferin's anticancer mechanisms, emphasizing its synergy with chemotherapeutic agents.

In one clinical trial, Ziaei et al. revealed the impact of inulin‐type fructans with varying degrees of polymerization on insulin resistance, blood lipids, anthropometric parameters, and hormonal profiles in overweight and obese women with PCOS. Biochemical and clinical outcomes were assessed at baseline and post‐intervention. Compared with the placebo group, participants in the HPI and OEI groups showed significant improvements in waist circumference, total testosterone, free androgen index, sex hormone‐binding globulin, and triglyceride levels (Ziaei et al. 2023).

In another clinical trial, the authors evaluated the analysis of fecal gut microbiota composition, utilizing partial 16S rRNA sequencing, biochemical and endocrine profiling, steatosis biomarkers (AST/ALT ratio), and anthropometric measurements. This revealed that after 180 days of supplementation, only the Novel Nutraceutical intervention group exhibited significant reductions in *Clostridium clostridioforme* (*Firmicutes phylum*), the *Firmicutes*/*Bacteroidetes* (F/B) ratio, and the De‐Ritis ratio. Concurrently, this group demonstrated increased abundances of *Bacteroides caccae* and *Bacteroides uniformis* (*Bacteroidetes phylum*) among sedentary overweight Brazilian participants (Nehmi‐Filho et al. 2024).

There are two comprehensive reviews and meta‐analyses covering the health implications of dietary fibers and bioactive carbohydrates from natural resources in this special e‐collection. This systematic review and dose–response meta‐analysis investigated the association between dietary fiber intake and chronic obstructive pulmonary disease (COPD) risk. Comprehensive searches were conducted across multiple databases (Scopus, Embase, and Medline) up to March 2023, identifying five prospective cohort studies involving 213,912 participants. COPD diagnosis predominantly relied on spirometry, the most widely utilized assessment method. Using a random‐effects model for data synthesis, both highest‐versus‐lowest intake comparisons and dose–response analyses were performed. Results demonstrated an inverse relationship between COPD risk and intake of total fiber (RR: 0.72; 95% CI: 0.64–0.80), cereal fiber (RR: 0.76; 95% CI: 0.68–0.86), and fruit fiber (RR: 0.75; 95% CI: 0.68–0.83), with no significant heterogeneity observed across studies (Valisoltani et al. 2023).

Another study evaluated the impact of purslane supplementation on lipid metabolism and inflammatory markers. Meta‐analysis revealed significant improvements in patients' lipid profiles compared with controls, with weighted mean differences (WMD) demonstrating reductions in serum triglycerides (−16.72 mg/dL; 95% CI: −22.49 to −10.96, *p* < 0.001), total cholesterol (−9.97 mg/dL; 95% CI: −19.86 to −0.07, *p* = 0.048), and C‐reactive protein levels (−1.22 mg/L; 95% CI: −1.63 to −0.80, *p* < 0.001). Furthermore, purslane intake significantly elevated high‐density lipoprotein cholesterol (HDL‐C) concentrations (WMD: +4.09 mg/dL; 95% CI: 1.77–6.41, *p* = 0.001), indicating favorable modulation of cardiometabolic risk factors. (Jafari et al. 2023).

Functional applications were also explored: Guar gum's versatility in food technology (Tahmouzi et al. 2023), chitosan‐polyvinyl alcohol coatings for egg preservation (Shurmasti et al. 2023), and konjac glucomannan's enhancement of plant protein gels (Yao et al. 2023). Thermal processing effects on pumpkin nutrition (Sıçramaz and Ayar 2023) and quinoa's role in glucose homeostasis (Ozcaliskan Ilkay et al. 2023) further expanded the scope.

In summary, the results of the above‐mentioned researches will bridge the gap between the knowledge on significant health implications of DFs and bioactive carbohydrates and their potential signaling and mechanisms. It also seeks to gather new knowledge and techniques for the identification and development of innovative adjuvants possessing health‐promoting attributes.

## Author Contributions

B.D. wrote the original manuscript. B.X. wrote the central part and improved this manuscript. All authors contributed to the article and approved the submitted version.

## Conflicts of Interest

The authors declare no conflicts of interest.

## Data Availability

The authors have nothing to report.
